# Drug–Drug Interactions of Selective Serotonin Reuptake Inhibitors: A Pharmacovigilance Study on Real-World Evidence from the EudraVigilance Database

**DOI:** 10.3390/ph17101278

**Published:** 2024-09-26

**Authors:** Carmen Maximiliana Dobrea, Adina Frum, Anca Butuca, Claudiu Morgovan, Laurentiu Stoicescu, Adriana Aurelia Chis, Anca Maria Arseniu, Luca Liviu Rus, Felicia Gabriela Gligor, Andreea Loredana Vonica-Tincu

**Affiliations:** 1 Preclinical Department, Faculty of Medicine, “Lucian Blaga” University of Sibiu, 550169 Sibiu, Romania; carmen.dobrea@ulbsibiu.ro (C.M.D.); claudiu.morgovan@ulbsibiu.ro (C.M.); adriana.chis@ulbsibiu.ro (A.A.C.); anca.arseniu@ulbsibiu.ro (A.M.A.); liviu.rus@ulbsibiu.ro (L.L.R.); felicia.gligor@ulbsibiu.ro (F.G.G.); loredana.vonica@ulbsibiu.ro (A.L.V.-T.); 2 Internal Medicine Department, Faculty of Medicine, “Iuliu Haţieganu” University of Medicine and Pharmacy, 400000 Cluj-Napoca, Romania; laurentiu.stoicescu@umfcluj.ro; 3 Cardiology Department, Clinical Municipal Hospital, 400139 Cluj-Napoca, Romania

**Keywords:** selective serotonin reuptake inhibitors, drug–drug interactions, inhibitory interaction, potentiating interaction, pharmacovigilance, real-world evidence

## Abstract

As the most common psychiatric symptom, depression represents a subject of high interest for the medical community. **Background/Objectives**: International guidelines consider selective serotonin reuptake inhibitors (SSRIs) the first-line treatment of depression. Although having better efficacy and tolerability in comparison to tricyclic antidepressants or monoamine oxidase inhibitors, the diversity and potential severity of adverse effects and interactions manifested by SSRIs, combined with the frequency of prescriptions, lead to the necessity of evaluating real-world data. The aim of this study was to identify and evaluate the drug interactions reported in EudraVigilance (EV) for the six SSRIs representatives that are authorized in Europe: fluoxetine (FXT), fluvoxamine (FVM), citalopram (CIT), escitalopram (ESC), paroxetine (PAR) and sertraline (SER). The entire class of SSRIs was examined as a comparator to identify whether one of the representatives was more prone to reporting. **Methods**: Descriptive analysis and disproportionality analysis were conducted on data extracted from the EV database. **Results**: A total of 326,450 adverse reactions (ADRs) were reported for the SSRIs group. Approximately a quarter of these (n = 83,201; 25.46%) were reported for SER and 22.37% (n = 73,131) for PAR. Of the total ADRs reported, 2.12% (n = 6925) represent preferred terms related to drug-drug interactions (DDIs): SER (n = 1474; 22.37%), CIT (n = 1272, 19.86), and FXT (n = 1309, 19.83%). Specific ADRs related to inhibitory activity represent 0.98%, and for potentiating activity, 1.89%. **Conclusions**: Although representing a small value of the total ADRs, DDIs may be related to severe outcomes. Awareness should be raised for this category of ADRs that can be reduced by the joined efforts of physicians and pharmacists.

## 1. Introduction

Mental health is an essential component of public health [1], its burden surpassing 16 trillion USD by 2030 [2]. Depression is the most common psychiatric symptom [3], affecting an estimated 5% of adults. The prevalence of depressive disorders has increased during the last decades, with accelerated growth during the recent pandemic, reaching approximately 280 million cases worldwide in 2023 [4]. Depression can negatively impact physical health [2] and the quality of life (QoL). As expected, the lowest QoL levels were reported before starting treatment. Although improved by the end of therapy, the QoL was lower in former depressive patients than in control groups [5]. Depression also increases the risk of suicide [6].

Pharmacological (selective serotonin reuptake inhibitors—SSRIs, tricyclic antidepressants, serotonin–norepinephrine reuptake inhibitors, monoamine oxidase inhibitors, and others) and nonpharmacological (psychological, behavioral therapies) approaches can be used for the treatment of depression [7].

SSRIs are considered the first-line treatment of depression by international guidelines [8,9,10]. Alongside efficacy, the safety of a medicinal product is important for improving health status. The mechanism of action of SSRIs is similar for all representatives: fluoxetine (FXT), fluvoxamine (FVM), citalopram (CIT), escitalopram (ESC), paroxetine (PAR), sertraline (SER), and it involves the inhibition of the serotonin transporter and implicitly the presynaptic reuptake of serotonin. A more recent study is inclined to also consider a cascading of events that lead to the downregulation of serotonin transporters in some areas of the brain and upregulation in others [11]. The pharmacokinetic and the pharmacodynamic properties are strongly influenced by the chemical structure. Interaction between SSRIs and several cytochrome P450 (CYP) isoenzymes are well documented. Worthy of note is the inhibition of CYP2D6 and CYP3A4 that may lead to clinical implications when SSRIs are administered together with drugs (e.g., propafenone, flecainide, tricyclic antidepressants, carbamazepine, cyclosporine, etc.) metabolized by the mentioned enzymes [12]. SSRIs are frequently well tolerated, and the management of overdoses has better outcomes in comparison to tricyclic antidepressants [13], although cardiac toxicity, expressed by ion channel activity interference, prolonged QT interval, and rhythm modifications, has been reported [14]. Serotoninergic syndrome (STS) is a rare, potentially fatal adverse effect triggered by high serotonin levels in the brain [11,14]. Summary product characteristics mention (i) adverse effects, with those classed as very common being insomnia, somnolence, nausea, dry mouth, and sexual dysfunctions, (ii) drug interactions, and (iii) warnings (e.g., increased suicidal risk) [15,16,17]. Adverse effects can be generated by exacerbated but similar mechanisms as the pharmacological properties.

Real-world evidence on adverse effects contributes to creating a comprehensive description of the risk–benefit ratio of a medicinal product. Pharmacovigilance systems, such as EudraVigilance (EV), continuously monitor and assess adverse drug reactions (ADRs) reported during clinical studies and the post-marketing era of medicinal products [18]. EV represents a reliable database set up by the European Medicines Agency (EMA) that is regularly updated. It comprises reports from the European Economic Area (EEA) and beyond, granting open access to both healthcare specialists and nonmedical users [19].

The diversity and potential severity of adverse effects and interactions manifested by SSRIs, combined with the frequency of prescriptions, lead to the necessity of evaluating real-world data and interpreting the results in the vast antidepressant therapeutic context.

The aim of this study was to identify and evaluate the drug interactions reported in EV for six SSRI representatives: FXT, FVM, CIT, ESC, PAR, and SER. The entire class of SSRIs was examined as a comparator to identify whether one of the representatives was more prone to reporting.

## 2. Results

### 2.1. Descriptive Analysis

According to data published in EV, ADRs were reported more frequently in the 18–64 years category: 47.59% (PAR)–58.24% (FVM). In the 65–84 years group, the frequency is between 10.19% (FXT) and 17.95% (CIT), and in the >85 years group, the frequency is between 1.71% (FXT) and 5.17% (CIT). There is a higher reporting frequency in the female group than in the male group: FVM (n = 2346, 54.94%)–ESC (n = 15,573, 62.69%). Most cases were reported from non-EEA, except CIT (41.8%) and ESC (42.84%). The higher frequency of cases reported from non-EEA is for FVM (n = 3039, 71.17%) (Table 1). Individual Case Reports (ICSRs) were submitted with a very high frequency by healthcare professionals (HPs): SER (n = 24,555; 68.37%) and FVM (n = 3746, 87.73%) (Table 1).

**Figure 1:**
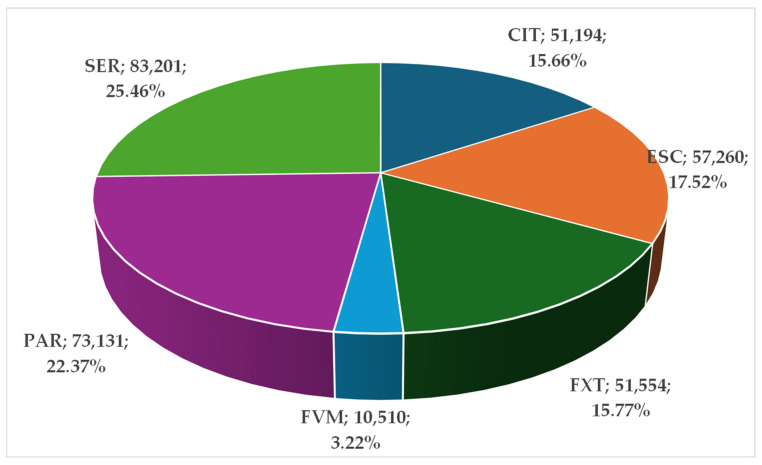
Total ADRs of SSRIs. CIT—citalopram; ESC—escitalopram; FXT—fluoxetine; FVM—fluvoxamine; PAR—paroxetine; SER—sertraline.

Figure 1 represents the distribution of ADRs produced by SSRIs. A total of 326,450 ADRs were reported for the SSRIs group. Approximately a quarter of these (n = 83,201; 25.46%) were reported for SER and 22.37% (n = 73,131) for PAR. CIT (n = 51,494; 15.66%), FXT (n = 51,554; 15.77%), and ESC (n = 57,260; 17.52%) have similar proportions in the total ADRs reported. The lowest proportion is presented for FVM (n = 10,510; 3.22%).

The one-way ANOVA test was performed to determine if there was a statistically significant difference between the reported ADRs in six SSRIs or 27 system organ classes (SOCs). The results show different *p*-values for the analyzed groups. The obtained value was *p* = 0.0027 (*p* < 0.05) for the six SSRIs and *p* < 0.0001 for the 27 SOCs; thus, statistically significant differences were determined for the analyzed data (Tables S2 and S3).

**Figure 2:**
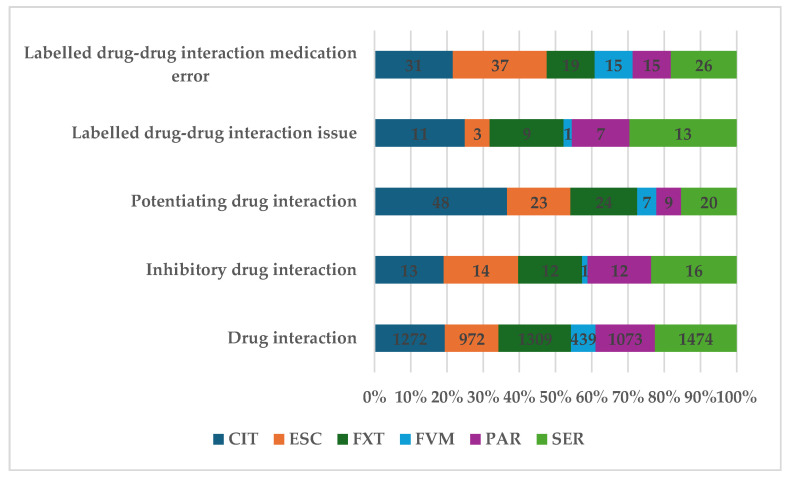
Distribution of ADRs by category of drug interaction. CIT—citalopram; ESC—escitalopram; FXT—fluoxetine; FVM—fluvoxamine; PAR—paroxetine; SER—sertraline.

Of the total ADRs reported, 2.12% (n = 6925) represent preferred terms (PTs) related to DDIs: SER (n = 1474; 22.37%), CIT (n = 1272, 19.86%), and FXT (n = 1309, 19.83%). Specific ADRs related to inhibitory activity represent 0.98% (n = 68), and for potentiating activity, 1.89% (n = 131). In the other reports, PTs do not specify the type of interaction (n = 6726, 97.13%) (Figure 2).

**Figure 3:**
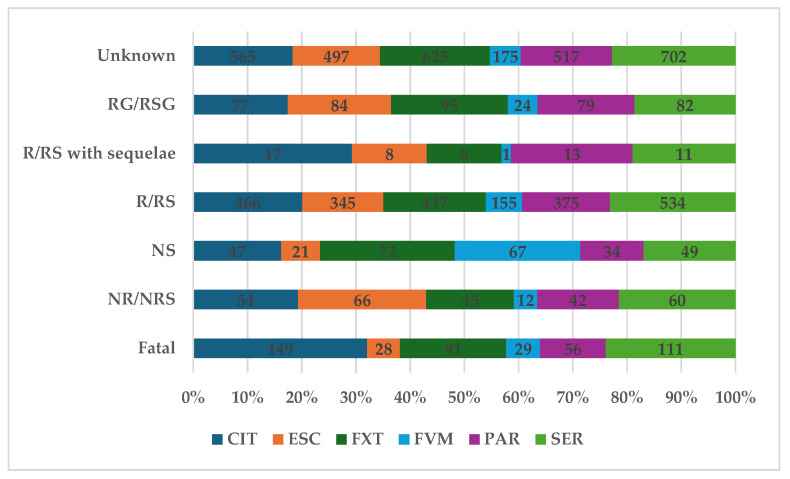
Distribution of ADRs by outcome. CIT—citalopram; ESC—escitalopram; FXT—fluoxetine; FVM—fluvoxamine; PAR—paroxetine; SER—sertraline; RG—recovering; RSG—resolving; R—recovered; RS—resolved; NS—not specified; NR—not recovered; NRS—not resolved.

According to Figure 3, 6.70% of total ADRs (n = 464) had a fatal outcome, and 4.00% (n = 279) were not recovered or resolved. The higher proportion of ADRs related to drug-drug interaction with a fatal outcome was registered for CIT (n = 149; 10.84%) and SER (n = 111; 7.17%), and the lowest for ESC (n = 28; 2.67%) and PAR (n = 56; 5.02%). Fortunately, for 33.4% of reports, the outcome was recovered or resolved, and the other 6.4% of the total were reported as recovering or resolving. The frequency of cases reported as recovered or resolved was between 31.83% (FXT, n = 437) and 34.47% (SER, n = 534).

### 2.2. Disproportionality Analysis

#### 2.2.1. All DDIs

**Figure 4:**
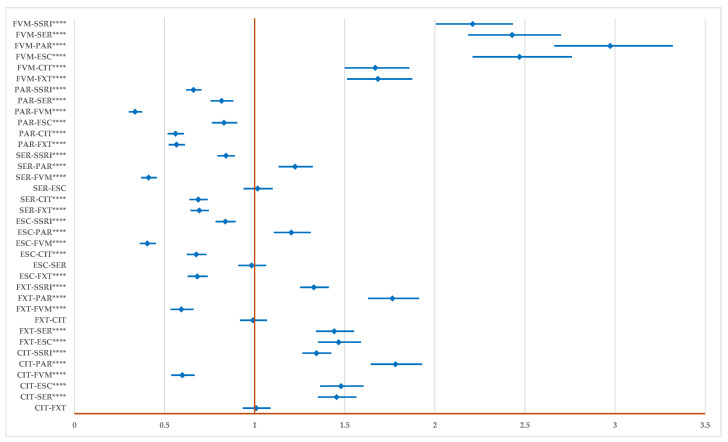
The disproportionality analysis of all signals related to total drug interactions. CIT—citalopram; ESC—escitalopram; FXT—fluoxetine; FVM—fluvoxamine; PAR—paroxetine; SER—sertraline; **** *p* ≤ 0.0001.

Figure 4 presents the disproportionality analysis of all signals related to DDIs. Compared to the group of all other SSRIs, for FVM (ROR: 2.21, 95% CI: 2.07–2.43), CIT (ROR: 1.34, 95% CI: 1.27–1.43), FXT (ROR: 1.33, 95% CI: 1.25–1.41) the reporting of ADRs related to DDIs is higher, and for ESC (ROR: 0.84, 95% CI: 0.78–0.90), SER (ROR: 0.84, 95% CI: 0.79–0.89), and PAR (ROR: 0.66, 95% CI: 0.62–0.71) is lower. Also, FVM, CIT (except in comparison to FVM), and FXT (except in comparison to FVM and CIT) present a higher probability of reporting. The probability of reporting ADRs for PAR is lower than for all drugs and for SER and ESC in comparison to CIT, FVM, and FXT.

#### 2.2.2. Drug Inhibition

**Figure 5:**
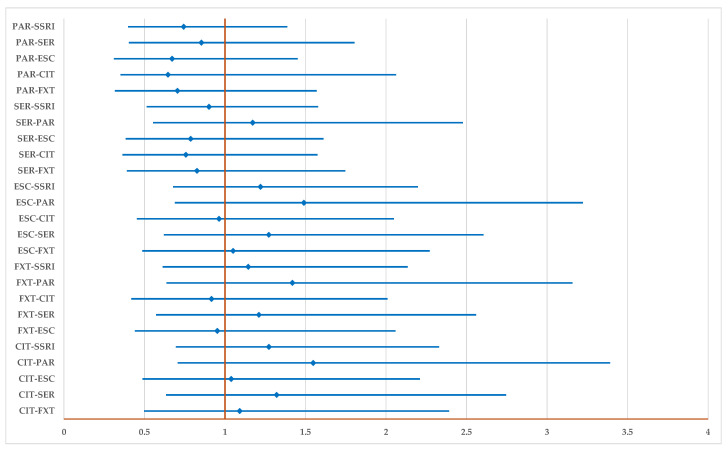
The disproportionality analysis of signals related to inhibitory drug interactions. CIT—citalopram; ESC—escitalopram; FXT—fluoxetine; FVM—fluvoxamine; PAR—paroxetine; SER—sertraline.

Figure 5 shows that PTs related to inhibitory interactions are not reported with a higher probability for any drug, including for the entire group of SSRIs.

#### 2.2.3. Potentiating Drug Interaction

**Figure 6:**
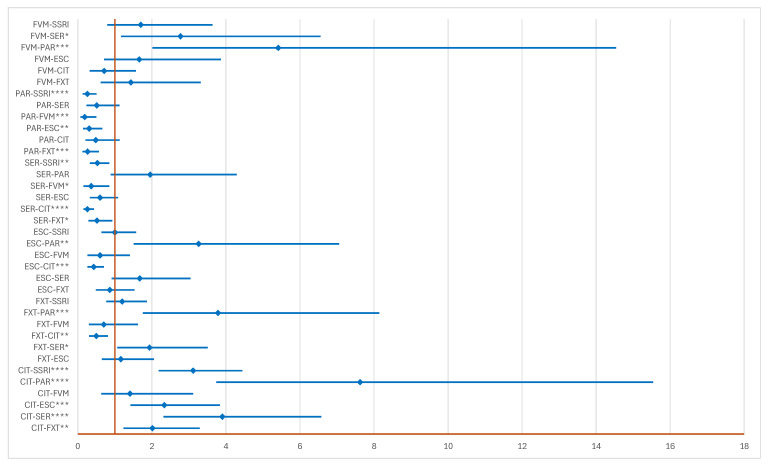
The disproportionality analysis of signals related to potentiating drug interactions. CIT—citalopram; ESC—escitalopram; FXT—fluoxetine; FVM—fluvamine; PAR—paroxetine; SER—sertraline; * *p* < 0.05; ** *p* ≤ 0.01; *** *p* ≤ 0.001; **** *p* ≤ 0.0001.

In the group of ADRs related to potentiating DDIs, SER (ROR: 0.53, 95% CI: 0.33–0.85) and PAR (ROR: 0.26, 95% CI: 0.13–0.50) have a lower probability of reporting than the entire class of SSRIs, and CIT (ROR: 3.12, 95% CI: 2.18–4.45) a higher probability. Also, the probability of reporting for CIT is higher than for all drugs except FVM. Compared to all drugs, SER, PAR, and ESC (except compared to PAR) do not have a higher probability of reporting (Figure 6).

#### 2.2.4. Unspecified DDIs

**Figure 7:**
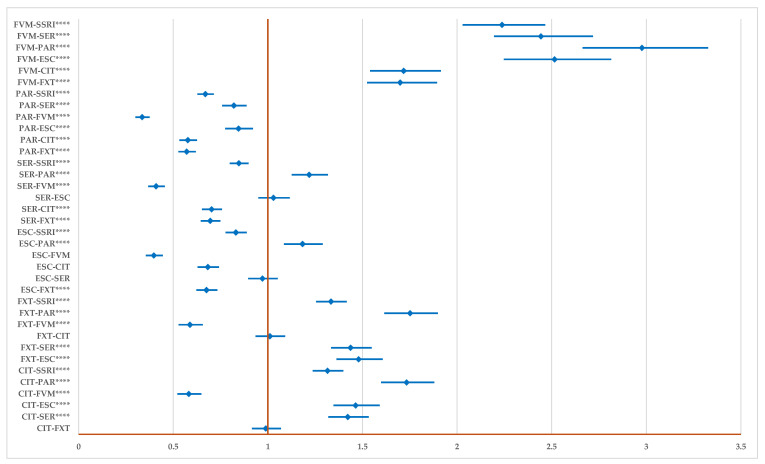
The disproportionality analysis of signals related to unspecified drug-drug interactions. CIT—citalopram; ESC—escitalopram; FXT—fluoxetine; FVM—fluvoxamine; PAR—paroxetine; SER—sertraline; **** *p* ≤ 0.0001.

Compared to the entire class of SSRIs, the probability of reporting ADRs related to DDIs is higher for CIT (ROR: 1.32; 95% CI: 1.24–1.40), FXT (ROR: 1.34, 95% CI: 1.26–1.42), and FVM (ROR: 2.24, 95% CI: 2.03–2.47). On the other hand, ESC (ROR: 0.83, 95% CI: 0.78–0.89), SER (ROR: 0.85, 95% CI: 0.80–0.90), and PAR (ROR: 0.67, 95% CI: 0.63–0.71) have a lower probability of reporting. FVM has a higher probability of reporting ADRs related to DDIs than all SSRIs, and PAR has a lower probability of reporting compared to all drugs. Also, a higher probability of reporting for SER (ROR: 1.22, 95% CI: 1.13–1.32) and ESC (ROR: 1.18, 95% CI: 1.09–1.29), except in comparison to PAR, could not be observed (Figure 7).

## 3. Discussion

The highest number of ADRs was registered for the 18–64 years age group (Table 1). This result may be related to the increased awareness of mental health nowadays [20,21,22], with the number of young people diagnosed with depression being reported as rising by various research groups [23,24,25]. Special attention is also attributed to postpartum and pregnancy-related depression [26]. The prevalence of postpartum depression reaches up to approximately 26%, depending on a number of factors such as individual health status and economic development level of origin country [27].

A notable difference is observed between genders; the number of ICSRs reported for women is approximately double the ones for men (Table 1) for all representatives of SSRIs. Two major factors may contribute to this result, one being the high prevalence of depression in women [28] and the second being the underdiagnosed depression and consequent lack of prescribed treatment in men [29].

A high number of ICSRs were registered from both the EEA and non-EEA regions (Table 1). The high prescription rate of SSRIs raised the concern of the scientific community about their quantification in sewage waters and their possible impact on the environment [30,31]. Five SSRIs were investigated: FXT, FVM, CIT, PAR, and SER, and detected in sewage water in Norway and the Arctic region [32]. Novel methods using zeolite proved effective for the removal of pharmaceuticals from wastewater [33].

SER (n = 83,201) and PAR (n = 73,131) had the most elevated number of ADRs, while the lowest values were related to FVM (n = 10,510) (Table S2). Between them, SER and PAR account for almost half of the total number of ADRs. The same duo was mentioned to have a variety of adverse effects, among which nausea, insomnia, sexual dysfunction, and others, by a large-scale meta-review on 80 medicines from the psychiatric therapeutic area [34] and a recent pharmacovigilance study [35]. Several studies were conducted on the safety and efficacy of SER, and a recent meta-analysis of clinical trials showed that the risk of adverse effects increases at values exceeding 150 mg [36]. The therapeutic interval for sertraline is 50–200 mg/day [37], so adverse effects may occur more frequently for the maximum allowed therapeutic dose. Also, the occurrence of ADRs caused by SER can contribute to treatment discontinuation [38]. To prevent the manifestation of adverse effects, the approach of using the minimal effective dose is recommended [34]. Even though SER is the SSRI with the highest number of reported ADRs, it continues to be acceptable for therapy and has a good safety profile, as shown by two recent meta-analyses [39,40].

PAR was linked in the scientific literature to a series of specific adverse effects, more often attributed to this molecule than to other SSRIs, such as weight gain and increased risk of congenital malformations [41,42]. Other studies showed that both SER and PAR can be linked to significant weight gain [43,44]. Overweight can cause multiple problems, and the early onset is detrimental to general health [45]; this determined the investigation on the influence of the drug on bodyweight in newborns when the mother is prescribed PAR and breastfeeding continues. The infant was not influenced in cases of daily doses of a maximum of 20 mg PAR [46]. A more recent report on congenital malformations caused by PAR considers previous findings as controversial and presents the case of a healthy infant of two years old with no malformation, although the mother heavily overdosed on PAR during the first trimester of pregnancy [47].

The total values of ADRs registered in EV for SSRIs as a class were the highest for the following SOC categories: Psychiatric disorders, Nervous system disorders, and General disorders and administration site conditions (Table S3). The top 10 categories are in line with data reported in the field [48,49].

Drug–drug interactions were reported in small amounts, constituting only 2% of all ADRs. The top three active pharmaceutical ingredients for which drug interactions were reported are SER, CIT, and FXT. SER also leads in this category, but CIT and FXT show higher values, although they did not distinguish themselves in other categories until now. Drug inhibition contributed to 1% of the total, and potentiating drug interactions were approximately double, contributing to approximately 1.9%. The patient information leaflet of SSRI representatives mentioned a number of drugs that have been proven to interact when administered at the same time. Multimorbid patients with complex therapies are at higher risk of DDIs [50]. An extensive review conducted by Sanchez et al. reported that PAR was considered to have more DDIs than SER or ESC due to its high capacity of inhibition of CYP2D6, while SER is mainly metabolized by CYP3A4 and CYP2C19, and ESC by CYP2C9 and CYP2C19 [51]. The low number of reports for DDIs found in EV by our study for FVM and ESC is expected and in line with the scientific literature [51,52]. PAR, FXT, and FVM also inhibit their own metabolism [53]. The fact that PAR is reported by many studies as having more frequent side effects and a high probability of DDIs may lead to its prescription in a lesser degree compared to other SSRIs and could offer an interpretation to the number of DDIs reports related to PAR found by this study. The influence of genetics on drug safety has been intensely researched recently and it offers answers to tailoring therapeutic doses depending on the speed of metabolization triggered by genetic polymorphism and epigenetic variability [54,55]. The use of established DDI checkers by both physicians and pharmacists was proven efficient in avoiding specific drug-drug associations when the risk of interacting is high [56].

About 10% of DDIs had an unfavorable result (6.7%—fatal, 4.0%—not recovered/not resolved) (Figure 3). For example, the inhibition of CYP2C19 by FXT can lead to the diminishing of efficacity for clopidogrel [35]; thus, the concomitant administration of these drugs was reported as fatal in some ICSRs. Moreover, STS is one of the severe adverse effects of drug interactions that increase serotonin levels in the brain and can be life-threatening when not managed properly [57]. The symptoms of STS include, but are not limited to, altered mental status, agitation, inducible (or spontaneous) muscular clonus, ocular clonus, akathisia, hyperreflexia, muscle rigidity, hypertension, and hyperthermia [58,59]. Over the years, STS has been described for all the representatives of SSRIs paired with a variety of other drugs: PAR and fentanyl [60], PAR and fentanyl, ondansetron, duloxetine and bupropion [61], PAR and clarithromycin [62], SER and linezolid [63], SER, quetiapine and trazodone [64], FXT, bupropion and dextromethorphan [65], FVM and mirtazapine [66], CIT and fentanyl [67], CIT and topiramate, CIT and cimetidine [68], CIT and fluconazole [69], ESC and miconazole [70], ESC and dextromethorphan [71].

The probability of reporting DDIs is higher for CIT, FVM, and FXT and lower for PAR, SER, and ESC. According to the results obtained by the disproportionality analysis using data provided in EV on 28 July 2024, PAR has the lowest probability of reporting drug interactions compared to all SSRIs as a class and to each representative. SER and ESC have a lower probability of reporting than FVM, FXT, CIT, and the whole SSRIs class (Figure 4). The real-life evidence of reports in EV shows a more complex status of DDIs than could have been expected from the scientific literature, where PAR emerged to be the SSRIs representative with most ADRs and the highest DDI probability [51].

For the inhibitory activity no significant differences between SSRIs were observed regarding the probability of reporting. This result could be related to the attitude the medical community expresses towards SSRIs, intervention measures being taken less than in the case of antibiotics [56]. Due to the capacity of specific SSRIs to inhibit various isoenzymes such as CYP1A2 (FVM), CYP2C9 and CYP2C19 (FVM, FXT), CYP2D6 (PAR, FXT) and CYP3A4 (FVM, FXT, SER, PAR) [72,73], it was expected to have less inhibitory interactions than potentiating interactions.

PAR and SER show a higher probability of reporting potentiating drug interactions but lower than the whole class of SSRIs. CIT shows a higher probability of reporting potentiating drug interactions, higher than each molecule (except FXT) and the whole class.

Taking into consideration all ADRs related to DDIs, FVM has a higher probability of reporting than each SSRI representative and the whole SSRI class, and PAR has a lower probability of reporting. This outcome may be influenced by the attention FVM received during the recent COVID-19 pandemic, with separate research groups considering it effective, more than other SSRIs, in reducing healthcare utilization in outpatients [74,75,76].

### Limitations of the Study

The limitations of the study are derived from the type of data available in established pharmacovigilance databases such as EV. This study includes the PTs related to drug interactions in the EV database. The results of the current descriptive analysis do not allow the precise identification of the causes that determine the high number of ADR reports for SER, one possible explanation being its more frequent prescription compared to other SSRIs. The disproportionality analysis indicates potential safety issues that might be reported and cannot be used to quantify the risk of ADRs for SSRIs. Open access data in the scientific literature offers limited information regarding the sales or prescription rates of SSRIs, and the existing ones lack homogeneity (the same active pharmaceutical ingredient can be prescribed for several illnesses, the age and gender of patients can vary, the time frame of the study is different, etc.) [77,78,79]. Pharmacovigilance databases such as EV do not store data regarding the exact number of SSRI doses that were prescribed and dispensed to patients, the access to this information is restricted.

The underreporting of ADRs is often an issue, with the total number of reports being constantly lower than the one of emerging ADRs. The reporting of ADRs depends on factors such as patient awareness, type of reaction, administration of other medicines, and conditions of use of the medication. Another limitation of the study is represented by general data collected in ICSRs; for example, the age is referred to only as an interval (e.g., 18–64 years category). Improvement in the quality of individual reports would be highly beneficial for future studies [80].

## 4. Materials and Methods

### 4.1. Study Design

A pharmacovigilance study regarding SSRI interactions was performed based on (ICSRs) submitted in the EV database (https://www.adrreports.eu/ (accessed on 1 August 2024)). All data submitted until 28 July 2024 were analyzed. Data were extracted between 29 July and 1 August 2024 from the “Number of Individual Cases for a Selected Reaction” tab. For the present study, (DDIs) were taken into consideration. Preferred terms (PTs) referring to interaction with food, herbs, alcohol, or tobacco were excluded.

### 4.2. Material

According to the Medical Dictionary for Regulatory Activities (MedDRA version 27.0), PTs referring to DDIs are included in “General disorders and administration site conditions” SOC (system organ classes). Thus, the PTs were grouped into three high-level terms: (1) unspecified drug interactions (drug interaction, labeled drug–drug interaction issue, and labeled drug–drug interaction medication error); (2) inhibitory drug interaction; (3) potentiating drug interaction. In the SSRIs category, CIT, ESC, FXT, FVM, PAR, and SER were identified.

### 4.3. Descriptive Analysis

A descriptive analysis of the general characteristics of ICSRs uploaded for all six SSRIs in the EV database until 28 July 2024 was performed. The characteristics of each report included information regarding the age category of the patient: 0–1 month, 2 months–2 years, 3–11 years, 12–17 years, 18–64 years, 65–85 years, more than 85 years, and not specified. Another characteristic of patients is represented by their sex (female, male, or not specified). ICSRs also contain information regarding the origin of reporters (EEA, non-EEA, not specified) and the qualification of the reporter (HP, non-HP, and not specified). Based on the data reported, was established the proportion of ADRs related to each SSRI. Subsequently, the distribution of ADRs by SOC in the sixth SSRI group and the distribution of ADRs for SSRIs in the 27th SOC group were compared. In the Supplementary Material, the percentage of the ADRs from each SOC in total reported for each SSRI is presented (Table S1). Another objective of the study was to present the distribution of ADRs by category of drug interaction: (1) unspecified drug interactions, (2) inhibitory drug interaction, and (3) potentiating drug interaction. The distribution of ADRs by outcome was also presented.

To assess the statistical significance of the analyzed data, the one-way ANOVA test was performed by using Microsoft Excel version 2407, Data Analysis Tool.

### 4.4. Disproportionality Analysis

To evaluate the signals reported as PTs in pharmacovigilance databases, disproportionality analysis shows the similarities and differences in reporting probability of ADRs. EMA recommended the calculation of reporting odds ratio (ROR) for the drug of interest compared to other drugs or all other drugs included in the database [81,82,83]. Also, the 95% confidence intervals (CIs) [84] were calculated. In the present study, we compared each SSRI one by one with all others, including with the entire group of all other SSRIs (resulting by summing of ADRs reported for the other SSRIs).

MedCalc Software Ltd. Odds ratio calculator [82] on https://www.medcalc.org/calc/odds_ratio.php (Version 20.123, accessed on 6 August 2024) was used to calculate ROR and 95% CI. A disproportionate signal is considered if a minimum of 5 cases are reported for each PT and if the lower limit of the 95% CI is higher than 1 [85].

### 4.5. Ethics

For descriptive and disproportionality analysis, respectively, ICSRs that include anonymous data were used. In this context, no ethical approval is required.

## 5. Conclusions

The aim of this study was to identify and evaluate the drug interactions reported in EV for the six SSRI representatives that are authorized in Europe. The entire class of SSRIs was examined as a comparator to identify whether one of the representatives is more prone to reporting than the others.

The highest numbers of DDIs were identified for SER, CIT, and FXT. The most frequent PT was “Drug interaction”. The potentiating interactions were reported in a higher number than the inhibitory drug interactions.

The higher proportion of ADRs related to DDIs with a fatal outcome was registered for CIT (n = 149; 10.84%) and SER (n = 111; 7.17%), and the lowest for ESC (n = 28; 2.67%) and PAR (n = 56; 5.02%). Fortunately, 33.4% of reports the outcome was recovered or resolved and the other 6.4% of the total were reported as recovering or resolving. The probability of reporting DDIs is higher for CIT, FVM, and FXT and lower for PAR, SER, and ESC. The limitations of the study were taken into consideration when interpreting the results.

DDIs may be related to severe outcomes, although they represent a small value of the total ADRs. Considering that polypharmacy increases the risk of DDIs, awareness should be raised for this category of ADRs that can be reduced by the joint efforts of physicians and pharmacists. Established DDI checkers should be developed and continuously employed in the health system.

## Figures and Tables

**Table 1:** General characteristics of ICSRs uploaded for SSRIs in the EV database until 28 July 2024. CIT—citalopram; EEA—European Economic Area; ESC—escitalopram; FXT—fluoxetine; FVM—fluvoxamine; HP—healthcare professional; NHP—non-HP; NS—not specified; PAR—paroxetine; SER—sertraline.

	CIT	ESC	FXT	FVM	PAR	SER
	n (%)	n (%)	n (%)	n (%)	n (%)	n (%)
Age category						
NS	3595	4499	4888	568	6909	7625
	(16.10%)	(18.11%)	(22.75%)	(13.30%)	(24.23%)	(21.23%)
0–1 months	400	487	443	56	1782	447
	(1.79%)	(1.96%)	(2.06%)	(1.31%)	(6.25%)	(1.24%)
2 months–2 years	58	64	77	18	78	84
	(0.26%)	(0.26%)	(0.36%)	(0.42%)	(0.27%)	(0.23%)
3–11 years	68	95	255	70	135	373
	(0.30%)	(0.38%)	(1.19%)	(1.64%)	(0.47%)	(1.04%)
12–17 years	413	738	1754	322	522	2185
	(1.85%)	(2.97%)	(8.16%)	(7.54%)	(1.83%)	(6.08%)
18–64 years	12,637	13,534	11,515	2487	13,574	18,738
	(56.58%)	(54.48%)	(53.58%)	(58.24%)	(47.59%)	(52.18%)
65–85 years	4010	4184	2190	665	4413	5288
	(17.95%)	(16.84%)	(10.19%)	(15.57%)	(15.47%)	(14.72%)
More than 85 years	1155	1239	368	84	1107	1173
	(5.17%)	(4.99%)	(1.71%)	(1.97%)	(3.88%)	(3.27%)
Sex category						
Female	13,762	15,573	13,207	2346	16,023	22,010
	(61.61%)	(62.69%)	(61.46%)	(54.94%)	(56.18%)	(61.29%)
Male	7741	8481	6144	1758	10,021	12,194
	(34.66%)	(34.14%)	(28.59%)	(41.17%)	(35.14%)	(33.95%)
NS	833	786	2139	166	2476	1709
	(3.73%)	(3.16%)	(9.95%)	(3.89%)	(8.68%)	(4.76%)
Origin						
EEA	12,993	14,198	9133	1231	12,034	17,842
	(58.17%)	(57.16%)	(42.50%)	(28.83%)	(42.19%)	(49.68%)
Non-EEA	9343	10,642	12,356	3039	16,480	18,071
	(41.83%)	(42.84%)	(57.50%)	(71.17%)	(57.78%)	(50.32%)
NS	0	0	1	0	6	0
	(0.00%)	(0.00%)	(0.00%)	(0.00%)	(0.02%)	(0.00%)
Reporter						
HP	17,485	18,263	16,402	3746	22,912	24,555
	(78.28%)	(73.52%)	(76.32%)	(87.73%)	(80.34%)	(68.37%)
Non-HP	4518	6492	4961	489	5399	10,972
	(20.23%)	(26.14%)	(23.09%)	(11.45%)	(18.93%)	(30.55%)
NS	333	85	127	35	209	386
	(1.49%)	(0.34%)	(0.59%)	(0.82%)	(0.73%)	(1.07%)

## Data Availability

Data contained within the article.

## Supplementary Materials

The following supporting information can be downloaded at https://www.mdpi.com/article/10.3390/ph17101278/s1, Table S1: Reported ADRs for SSRIs depending on SOCs; Table S2: One-Way ANOVA test results for the reported ADRs (for the twenty-seventh SOCs) in relation to the six SSRIs. CIT—citalopram; ESC—escitalopram; FXT—fluoxetine; FVM—fluvoxamine; PAR—paroxetine; SER—sertraline; Table S3: One-Way ANOVA test results for the reported ADRs (for the six SSRIs) in relation to the twenty-seventh SOCs.
