# Supplementary material for: Drug–Drug Interactions of Selective Serotonin Reuptake Inhibitors: A Pharmacovigilance Study on Real-World Evidence from the EudraVigilance Database
Source: Pharmaceuticals (Basel). 2024 Sep 26;17(10):1278. doi: 10.3390/ph17101278 (PMC11510210; doi:10.3390/ph17101278)
Supplement: Supplementary file 1 [file pharmaceuticals-17-01278-s001.zip › Supplementary material.pdf]

SUPPLEMENTARY MATERIAL

Table S1. Reported ADRs for SSRIs depending on SOCs

	CIT		ESC		FXT		FVM		PAR		SER	
**SOC**	**n**	**%**	**n**	**%**	**n**	**%**	**n**	**%**	**n**	**%**	**n**	**%**
**Blood and lymphatic system disorders**	787	1.5%	736	1.3%	698	1.4%	194	1.8%	897	1.2%	882	1.1%
**Cardiac disorders**	2545	5.0%	2204	3.8%	2195	4.3%	400	3.8%	2928	4.0%	2909	3.5%
**Congenital, familial and genetic disorders**	497	1.0%	602	1.1%	1519	2.9%	40	0.4%	3997	5.5%	1700	2.0%
**Ear and labyrinth disorders**	413	0.8%	602	1.1%	335	0.6%	54	0.5%	848	1.2%	896	1.1%
**Endocrine disorders**	312	0.6%	473	0.8%	313	0.6%	77	0.7%	608	0.8%	633	0.8%
**Eye disorders**	875	1.7%	1023	1.8%	881	1.7%	171	1.6%	1378	1.9%	1758	2.1%
**Gastrointestinal disorders**	3309	6.5%	4314	7.5%	2898	5.6%	662	6.3%	4594	6.3%	7341	8.8%
**General disorders and administration site conditions**	6006	11.7%	6513	11.4%	5785	11.2%	1,313	12.5%	8000	10.9%	9605	11.5%
**Hepatobiliary disorders**	406	0.8%	588	1.0%	542	1.1%	202	1.9%	938	1.3%	965	1.2%
**Immune system disorders**	328	0.6%	324	0.6%	351	0.7%	29	0.3%	219	0.3%	511	0.6%
**Infections and infestations**	714	1.4%	760	1.3%	786	1.5%	155	1.5%	1026	1.4%	1354	1.6%
**Injury, poisoning and procedural complications**	5439	10.6%	5291	9.2%	6355	12.3%	887	8.4%	8481	11.6%	7544	9.1%
**Investigations**	2920	5.7%	3198	5.6%	2853	5.5%	937	8.9%	3909	5.3%	4123	5.0%
**Metabolism and nutrition disorders**	2277	4.4%	2428	4.2%	1777	3.4%	360	3.4%	2663	3.6%	2901	3.5%
**Musculoskeletal and connective tissue disorders**	1456	2.8%	1770	3.1%	1435	2.8%	281	2.7%	2095	2.9%	2827	3.4%
**Neoplasms benign, malignant and unspecified (incl cysts and polyps)**	157	0.3%	186	0.3%	277	0.5%	18	0.2%	252	0.3%	484	0.6%
**Nervous system disorders**	6928	13.5%	8318	14.5%	6416	12.4%	1,825	17.4%	9834	13.4%	11510	13.8%
**Pregnancy, puerperium and perinatal conditions**	477	0.9%	532	0.9%	961	1.9%	45	0.4%	812	1.1%	800	1.0%
**Product issues**	163	0.3%	329	0.6%	243	0.5%	25	0.2%	351	0.5%	517	0.6%
**Psychiatric disorders**	8127	15.9%	8802	15.4%	7982	15.5%	1,528	14.5%	9696	13.3%	12052	14.5%

	CIT		ESC		FXT		FVM		PAR		SER	
**SOC**	**n**	**%**	**n**	**%**	**n**	**%**	**n**	**%**	**n**	**%**	**n**	**%**
**Renal and urinary disorders**	639	1.2%	981	1.7%	746	1.4%	212	2.0%	1124	1.5%	1220	1.5%
**Reproductive system and breast disorders**	850	1.7%	1185	2.1%	719	1.4%	110	1.0%	986	1.3%	1637	2.0%
**Respiratory, thoracic and mediastinal disorders**	1780	3.5%	1639	2.9%	1926	3.7%	308	2.9%	2491	3.4%	2887	3.5%
**Skin and subcutaneous tissue disorders**	1969	3.8%	2383	4.2%	1818	3.5%	344	3.3%	2574	3.5%	3471	4.2%
**Social circumstances**	258	0.5%	391	0.7%	274	0.5%	25	0.2%	507	0.7%	533	0.6%
**Surgical and medical procedures**	247	0.5%	318	0.6%	258	0.5%	35	0.3%	340	0.5%	356	0.4%
**Vascular disorders**	1315	2.6%	1370	2.4%	1211	2.3%	273	2.6%	1583	2.2%	1785	2.1%

Table S2. One-Way ANOVA test results for the reported ADRs (for the twenty-seventh SOCs) in relation to the six SSRIs. CIT – citalopram ; ESC – escitalopram; FXT – fluoxetine; FVM – fluvoxamine; PAR – paroxetine; SER – sertraline.

SSRI	Total number of SOCs	Sum of ADRs	Average of ADRs	P-value
CIT	27	51,194	1896.074	0.0027
ESC	27	57,260	2120.741	
FXT	27	51,554	1909.407	
FVM	27	10,510	389.2593	
PAR	27	73,131	2708.556	
SER	27	83,201	3081.519	

Table S3. One-Way ANOVA test results for the reported ADRs (for the six SSRIs) in relation to the twenty-seventh SOCs.

SOC	Total number of SSRIs	Sum of ADRs	Average of ADRs	P-Value
Blood and lymphatic system disorders	6	4194	699.00	<0.0001
Cardiac disorders	6	13,181	2196.83	
Congenital, familial and genetic disorders	6	8355	1392.50	
Ear and labyrinth disorders	6	3148	524.67	
Endocrine disorders	6	2416	402.67	
Eye disorders	6	6086	1014.33	
Gastrointestinal disorders	6	23,118	3853.00	
General disorders and administration site conditions	6	37,222	6203.67	
Hepatobiliary disorders	6	3641	606.83	
Immune system disorders	6	1762	293.67	
Infections and infestations	6	4795	799.17	
Injury, poisoning and procedural complications	6	33,997	5666.17	

SOC	Total number of SSRIs	Sum of ADRs	Average of ADRs	P-Value
Investigations	6	17,940	2990.00	<0.0001
Metabolism and nutrition disorders	6	12,406	2067.67	
Musculoskeletal and connective tissue disorders	6	9864	1644.00	
Neoplasms benign, malignant and unspecified (incl cysts and polyps)	6	1374	229.00	
Nervous system disorders	6	44,831	7471.83	
Pregnancy, puerperium and perinatal conditions	6	3627	604.50	
Product issues	6	1628	271.33	
Psychiatric disorders	6	48,187	8031.17	
Renal and urinary disorders	6	4922	820.33	
Reproductive system and breast disorders	6	5487	914.50	
Respiratory, thoracic and mediastinal disorders	6	11,031	1838.50	
Skin and subcutaneous tissue disorders	6	12,559	2093.17	
Social circumstances	6	1988	331.33	
Surgical and medical procedures	6	1554	259.00	
Vascular disorders	6	7537	1256.17	
